# Acupuncture for patients with insomnia disorder using resting-state functional magnetic resonance imaging: a protocol for a randomized controlled trial

**DOI:** 10.1186/s13063-019-3836-z

**Published:** 2019-12-09

**Authors:** Jing Guo, Siyi Yu, Chunhong Liu, Guiling Wang, Bin Li

**Affiliations:** 1 Acupuncture and Moxibustion Department, Beijing Hospital of Traditional Chinese Medicine, Capital Medical University, 23 Art Museum Backstreet, Dongcheng District, Beijing, 100010 China; 2 Chengdu University of Traditional Chinese Medicine, 37 Shi’er Qiao Road, Jinniu District, Chengdu, 610072 Sichuan China; 3 Beijing Hospital of Traditional Chinese Medicine, Capital Medical University, Beijing Institute of Traditional Chinese Medicine, 23 Meishuguanhou Street, Dongcheng District, Beijing, 100010 China

**Keywords:** Acupuncture, insomnia disorder, Resting-state functional magnetic resonance imaging study

## Abstract

**Background:**

Insomnia is among the most prevalent of the sleep-related disorders. Insomnia disorder is associated with a brain hyperarousal state manifested by abnormal regional brain activity and resting state functional connectivity. Acupuncture improves sleep quality and modulates the hyperarousal state; however, the underlying neurobiological basis for improved sleep quality is poorly understood. The purpose of this clinical trial is to study the efficacy of acupuncture in the treatment of insomnia disorder. In addition, the neural mechanism by which acupuncture affects insomnia disorder will be explored using resting-state functional magnetic resonance imaging (rs-fMRI) and neuropsychological parameters.

**Methods and design:**

A randomized, patient- and assessor-blinded trial will be conducted. We will randomize (in a 1:1 ratio) 60 eligible patients with insomnia disorder into a real acupuncture group or a sham acupuncture group. Interventions will be administered three times per week over a 4-week period, with an 8-week follow-up period. The healthy control group will consist of 30 age- and sex-matched healthy individuals who sleep well without any treatment intervention.

All participants will undergo neuropsychological and rs-fMRI evaluations. The change in Pittsburgh Sleep Quality Index (PSQI) scores is the primary outcome parameter. The secondary outcome parameters include the Hyperarousal scale (HAS), rs-fMRI measurements, the Fatigue scale-14 (FS-14), the Hamilton depression scale (HAMD), the Hamilton anxiety scale (HAMA), a sleep diary, and an actigraph.

Assessment of all parameters will be performed at baseline, post-treatment, and during follow-up. Analyses will be implemented based on intention-to-treat.

**Discussion:**

The study results will be used to clarify the effectiveness and elucidate the mechanism by which acupuncture improves sleep quality in patients with insomnia disorder.

**Trial registration:**

Chinese Clinical Trials Register, ChiCTR1800015282. Registered on 20 March 2018.

## Background

Insomnia, which affects 10 to 15% of the population [1], is characterized by problems falling asleep, remaining asleep, or obtaining refreshing sleep, and is accompanied by impairments of daytime functioning [2]. However, the pathophysiology of insomnia is still under investigation. The most compelling hypothesis for insomnia is the hyperarousal model [3], which states that somatic, cognitive, and brain activation are increased in patients with insomnia [4].

The first-line therapies for insomnia are pharmacologic intervention with hypnotic medications and cognitive behavioral therapy. However, many patients are troubled by the side effects of medications, including lingering daytime sedation, cognitive impairment, and medication dependence. Psychotherapy is time-consuming and depends on the availability of trained therapists [5]. These factors lead to a low compliance rate for insomnia therapy [6]. Therefore, some patients have sought alternative treatments. Acupuncture, a critical component of traditional Chinese medicine, may relieve the symptoms of insomnia [7–10]. Nevertheless, the underlying neural basis for the effects of acupuncture remains largely unknown.

Recently, neuroimaging investigators have become interested in the spontaneous low-frequency fluctuations in brain activity when at rest. Studying these low-frequency fluctuations may help in elucidating the brain’s intrinsic functional organization to aid in understanding the neurobiological mechanisms of insomnia and its treatment [11]. For instance, enhanced functional connectivity between the amygdala and the premotor and sensorimotor cortex occurs in patients with insomnia disorder. The amygdala is closely related to fears, depression, and anxieties. Therefore, insomnia disorder may be related to increased negative emotional memory activity [12]. The insula is connected to other parts of the emotional circle [13], and anterior insula interactions with salient networks have been detected [14]. In a study by Dong, increased whole brain positive connectivity detected in patients with insomnia disorder was associated with a hyperarousal state [15]. Furthermore, regional homogeneity (ReHo) increased in the left insula of patients with insomnia disorder that positively correlates with Self-Rating Anxiety Scale scores [16]. Taken together, research using data from resting-state functional magnetic resonance imaging (rs-fMRI) suggests a brain hyperarousal state, especially in the emotion-related areas. Thus, patients with insomnia may benefit from modulation of this excessive brain activity. A recent fMRI study indicates that acupuncture can modulate the activity of the cerebro-cerebellar and limbic systems [17].

The aims of this clinical trial are: 1) to determine the effectiveness of acupuncture on sleep quality in patients with insomnia disorder using neuropsychological and rs-fMRI methods; and 2) to elucidate the neurological mechanism by which acupuncture improves sleep quality. In addition to rs-fMRI, actigraphy will also be used to monitor sleep quality by assaying time in bed, sleep latency, waking after sleep onset, sleep time, and sleep efficiency. Patients will be asked to complete sleep diaries and self-report questionnaires, such as the Pittsburgh Sleep Quality Index (PSQI), Hyperarousal scale (HAS), Hamilton Depression Scale **(**HAMD), Hamilton anxiety scale (HAMA), and Fatigue scale-14 (FS-14).

## Methods and design

### Objectives

The study objectives are: 1) to determine the efficacy of acupuncture for the treatment of insomnia disorder; and 2) to elucidate the neural mechanisms for the effects of acupuncture on insomnia disorder.

### Hypotheses

1. We hypothesize that sleep quality will be significantly improved in the group receiving acupuncture treatment compared with the sham group, as assessed by both subjective (PSQI) and objective (actigraphy) measurements.
2. The hyperarousal state, measured by HAS, will be decreased more in the group receiving acupuncture than the sham group.
3. Based on fMRI examination, functional connectivity and ReHo in the emotion-related areas will be altered in the group receiving acupuncture compared with the sham group.

### Setting

The trial site will be the Acupuncture and Moxibustion Department of the Beijing Hospital of Traditional Chinese Medicine, Capital Medical University, Beijing, China. Sixty patients with insomnia disorder will be assigned to either a real acupuncture group or a sham group, using a digital randomization table in a 1:1 ratio.

### Trial design

**Fig. 1:**
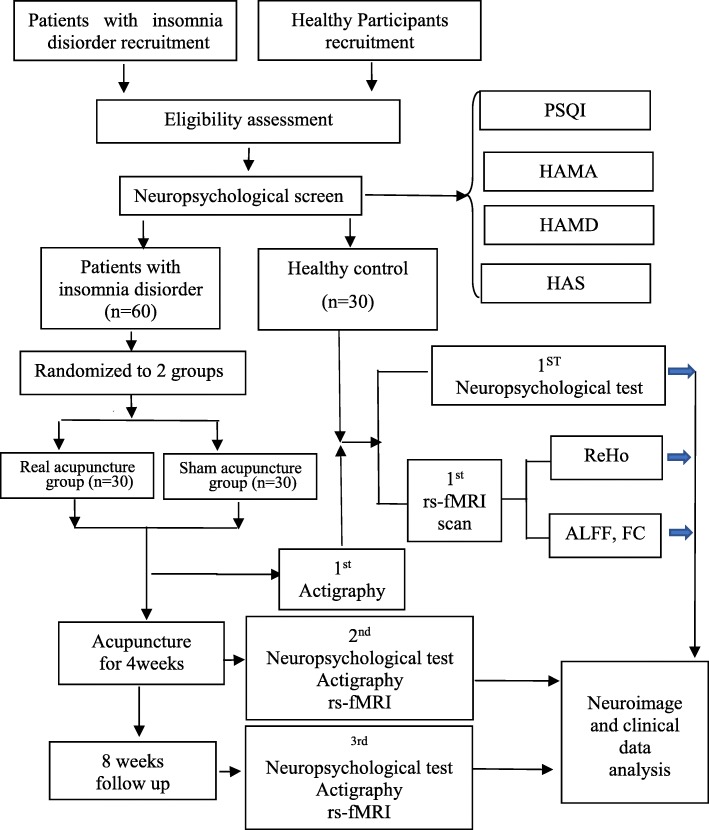
Flow chart of study. ALFF amplitude of low-frequency fluctuation, FC functional connectivity, FS-14 Fatigue scale-14, HAMA Hamilton Anxiety scale, HAMD Hamilton Depression scale, HAS Hyperarousal scale, PSQI Pittsburgh Sleep Quality Index, ReHo regional homogeneity, rs-fMRI resting-state functional magnetic resonance imaging

This study will be conducted as a single-center, prospective parallel group, patient- and assessor-blinded, randomized controlled trial. The trial will be conducted in accordance with the Consolidated Standards of Reporting Trials [18] and Standards for Reporting Interventions in Clinical Trials of Acupuncture principles [19] to fulfill adequate reporting standards for randomized controlled trials. Sixty patients with insomnia disorder will be assigned to either a real acupuncture group or a sham acupuncture group, using a digital randomization table in a 1:1 ratio. Participants will keep a sleep diary at baseline, during week 4 of treatment, and during week 8 of the follow-up period. Both the neuropsychological measurements and fMRI scans will be performed at baseline, after completion of the acupuncture treatments, and at the conclusion of the follow-up period. Thirty age- and sex-matched healthy individuals who sleep well will be recruited as controls for the fMRI data analysis. All outcome measures will be performed once for the healthy control patients (Fig. 1).

### Participants and recruitment

Posted notices in the hospital and recruitment information on the website of the Beijing Hospital of Traditional Chinese Medicine will be used to recruit study participants.

Participants will be informed about the benefits, as well as possible risks, of participation in the study, including poor clinical outcome and adverse events associated with acupuncture therapy. Completion of consent forms will be compulsory before trial participation and randomization. On the consent form, participants will be asked if they agree to the use of their data should they choose to withdraw from the trial. Participants will also be asked for permission for the research team to share relevant data with people from the hospital taking part in the research or from regulatory authorities. This trial does not involve collecting biological specimens for storage. The model consent form and other related documentation given to participants are available from the corresponding author on request.

A neurologist will determine the PSQI, HAS, HAMD and HAMA scores to decide whether the participants meet the inclusion criteria. Patients who satisfy the inclusion criteria will be randomized (1:1) into either a real acupuncture group or a sham acupuncture group. All participants will complete a baseline rs-fMRI, a 3-day actigraphy test, and a 1-week sleep diary. Participants will receive acupuncture treatment for 4 weeks. In addition, 30 age- and sex-matched healthy individuals who sleep well will be recruited as healthy controls for the fMRI data analysis. All personal information about the participants will be kept confidential by the research investigators and used for research purposes only.

### Inclusion criteria for patients with insomnia disorder

Compliance with the following criteria will be required for participation in the study:

1. Aged between 20 and 60 years
2. Meeting the diagnostic criteria for insomnia disorder according to the Diagnostic and Statistical Manual of Mental Disorders, Fifth Edition
3. A PSQI score >8 points; HAMD score <7; HAMA score <14; and HAS score >32
4. A signed informed consent form specific for this trial
5. Have not received medications for anxiety, depression, or insomnia within 1 month prior to enrollment in the study

### Exclusion criteria for patients with insomnia disorder

Patients will be excluded from enrollment in the trial if they meet one or more of the following:

1. Diagnosis of anxiety, depression, or schizophrenia
2. Diagnosis of severe medical disorders, including cardiac, cerebral, renal, hepatic, or metabolic diseases, or benign prostatic hyperplasia
3. Diagnosis of other sleep disorders, such as sleep apnea and restless leg syndrome
4. Women who are pregnant or breastfeeding
5. Difficulty completing the inspection and treatment
6. Contraindication for MRI including claustrophobia; abnormal signal or obvious asymmetrical head structure by MRI
7. Acupuncture therapy for insomnia treatment in the past month
8. Addiction to drugs or alcohol

### Inclusion criteria for healthy controls

1. No complaints about sleep quality and quantity
2. Aged between 20 and 60 years of age
3. A signed informed consent form specific for this trial
4. Have not received medications for anxiety, depression, or insomnia within 1 month prior to enrollment in the study

### Exclusion criteria for healthy controls

1. Diagnosis of anxiety, depression, or schizophrenia
2. Diagnosis of severe medical disorders, including cardiac, cerebral, renal, hepatic, or metabolic diseases, or benign prostatic hyperplasia
3. Diagnosis of other sleep disorders, such as sleep apnea and restless leg syndrome
4. Women who are pregnant or breastfeeding
5. Difficulty completing the inspection
6. Contraindications for MRI including claustrophobia; abnormal signal or obvious asymmetrical head structure by MRI
7. Acupuncture therapy in the past month
8. Addiction to drugs or alcohol

### Sample size

This study aims to determine the efficacy and central mechanism by which acupuncture affects insomnia disorder. Based on our previous pilot study [20], the PSQI score significantly decreased by 4.43 ± 3.60 in the group treated with acupuncture compared to 1.30 ± 2.58 in the control group. Based on a power analysis, 26 patients per group were required to detect a significant difference (power = 0.9, α = 0.05, two-sided). Thus, we plan on recruiting 30 patients per group to compensate for a 15% drop out rate. There is no known sample size calculation for fMRI research. However, for the exploratory study, 15 to 30 patients are adequate to test the null hypothesis [21, 22].

### Ethics

Protocol approval was obtained by the Medical Ethical Committee of Beijing Hospital of Traditional Chinese Medicine on 12 March 2018 (2018BL-002-02). Written informed consent will be attained from each participant. This trial is funded by the National Natural Science Foundation of China (81,774,391, 81,871,507).

### Randomization, allocation concealment, and blinding

After meeting the eligibility requirements, participants will be randomly assigned (in a 1:1 ratio) to the real or the sham acupuncture group. The random allocation sequence will be generated by an independent statistician using Statistical Analysis Software (version 9.3, SAS Institute Inc., Cary, NC, USA). The randomization list will be closed in computer-generated opaque envelopes with sequence numbers printed on the outside of the envelopes. After the patient has been screened, is determined to be eligible, and has given informed consent, the envelopes will be opened by the researchers.

The acupuncturists cannot be blinded and therefore will be excluded from assessments and data processing. In the trial, the participants, data analysts, and outcome assessors will be blinded. Assessors will not perform data analysis. Unblinding should only be performed in case of an emergency, such as any serious adverse events.

In addition, sterile steel needles of the same size and number for each treatment session will be used in both groups. Participants will be required to write down if they have received real acupuncture at the final treatment. The answer ‘Y’ or ‘N’ will be assessed to determine the confidence in the treatment.

### Interventions

#### Real acupuncture group (shown in Fig. 2 and Table 1)

The selection of acupoints is based on the experience of experts and our previous clinical research [23]. All acupuncturists will receive training to ensure consistent acupuncture technique on all participants. Acupoints on the vertex, including Baihui (GV-20), Shenting (GV-24), Benshen (GB-13), and Sishencong (EX-HN1), will be inserted horizontally for 10 mm. Sanyinjiao (SP-6) will be inserted for 10 mm straight in, while Neiguan (PC-6) and Shenmen (HT-7) will be inserted perpendicularly for 5 mm. Needle manipulation, such as needling rotation, lifting, and inserting, will be employed to attain the ‘De Qi’ sensation (swelling, pain, numbness, distention, and heaviness). Needles will be kept in position for 30 min. Stainless steel needles used in the trial will be 0.32 mm × 40 mm. The real acupuncture therapy will be performed three times per day at 2-day intervals for 4 weeks. The localization of the acupoints will be in accordance with the World Health Organization standard acupuncture locations [24].

**Fig. 2:**
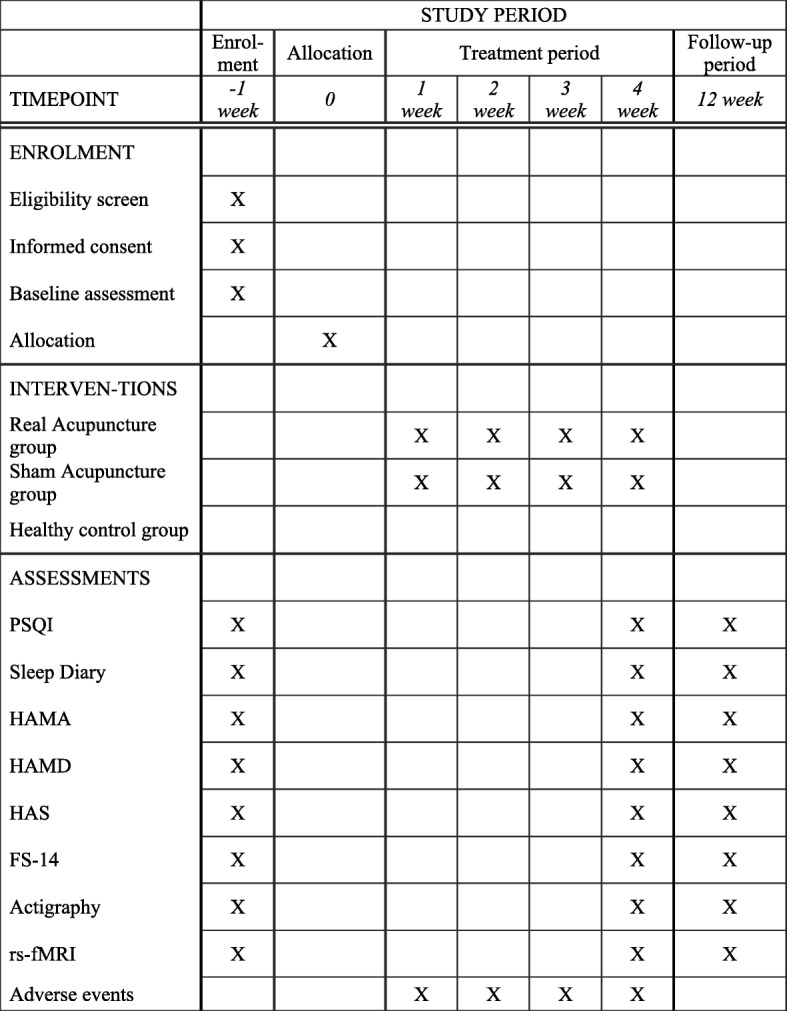
Enrollment schedule, treatment, and outcome measures. FS-14 Fatigue scale-14, HAMA Hamilton Anxiety scale, HAMD Hamilton Depression scale, HAS Hyperarousal scale, PSQI Pittsburgh Sleep Quality Index, rs-fMRI resting state functional magnetic resonance imaging

**Table 1:** Locations and manipulations of real and sham acupuncture

Group	Acupoints	Locations	Needle insertion	Needle manipulation
Real acupuncture group	Baihui (GV-20)	On the head, 5B-cun superior to the anterior hairline, on the anterior median line	Oblique needle insertion at depth of 10 mm	Manipulation and De-qi sensation felt by practitioner and patient
	Shenting (GV-24)	On the head, 0.5B-cun superior to the anterior hairline, on the anterior median line		
	Benshen (GB-13)	On the head, 0.5B-cun superior to the anterior hairline, 3B-cun lateral to the anterior median line		
	Sishencong (EX-HN-1)	Four points at the vertex of the scalp, grouped around Baihui GV-20 and located 1 cun anterior, posterior and lateral to it		
	Neiguan (PC-6)	On the anterior aspect of the forearm, between the tendons of the palmaris longus and the flexor carpi radialis, 2B-cun proximal to the palmar wrist crease	Perpendicular needle insertion at depth of 5 mm	
	Shenmen (HT-7)	On the anteromedial aspect of the wrist, radial to the flexor carpi ulnaris tendon, on the palmar wrist crease		
	Sanyinjiao (SP-6)	On the tibial aspect of the leg, posterior to the medial border of the tibia, 3B-cun superior to the prominence of the medial malleolus	Straight needle insertion at depth of 10 mm	
Sham acupuncture group	Binao (LI-14),	On the lateral aspect of the arm, just anterior to the border of the deltoid muscle, 7B-cun superior to LI-11	Superficial needle insertion at depth of 1 mm	No manual stimulation and De qi sensation
	Shousanli (LI-10)	On the posterolateral aspect of the forearm, on the line connecting LI-5 with LI-11, 2B-cun inferior to the cubital crease		
	Yangchi (TE-4)	On the posterior aspect of the wrist, in the depression ulnar to the extensor digitorum tendon, on the dorsal wrist crease		
	Waiguan (TE-5)	On the posterior aspect of the forearm, midpoint of the interosseous space between the radius and the ulna, 2B-cun proximal to the dorsal wrist crease		
	Fengshi (GB-31)	On the lateral aspect of the thigh, in the depression posterior to the iliotibial band where the tip of the middle finger rests, when standing up with the arms hanging alongside the thigh		
	Liangqiu (ST-34)	On the anterolateral aspect of the thigh, between the vastus lateralis muscle and the lateral border of the rectus femoris tendon, 2B-cun superior to the base of the patella		
	Futu (ST-32)	On the anterolateral aspect of the thigh, on the line connecting the lateral end of the base of the patella with the anterior superior iliac spine, 6B-cun superior to the base of the patella		

#### Sham acupuncture group (shown in Fig. 2 and Table 1)

The sham acupuncture group will be given sham therapy with the same treatment duration and frequency of sessions as the real acupuncture group. Based on previous clinical experience, we chose acupoints that are ineffective in treating insomnia for the sham acupuncture group. The following acupoints will be punctured in the sham group: Binao (LI-14), Shousanli (LI-10), Yangchi (TE-4), Waiguan (TE-5), Fengshi (GB-31), Liangqiu (ST-34), and Futu (ST-32). The same needle size as in the real acupuncture group will be used. Unlike the real acupuncture application, needles will be inserted superficially 1 mm without manual stimulation. The needles will be kept in place for 30 min. Meanwhile, creation of the De qi sensation will be prevented by not manipulating the needles.

#### Healthy control group

No acupuncture intervention will be conducted in the healthy control group.

### Functional MRI scanning procedure

The rs-fMRI data will be obtained with a Siemens TRio 3.0 Tesla MRI scanner in Beijing Hospital of Traditional Chinese Medicine. All rs-fMRI images will be taken from a contiguous echo planar imaging template under the following conditions: 33 axial slices, repetition time = 2000 ms, echo time = 30 ms, thickness/gap = 3.5/0.6 mm, field of view = 220 × 220 mm^2^, flip angle = 90°, and matrix size 64 mm × 64 mm with 240 volumes. All participants will receive the following instructions: stay awake, do not move, close your eyes, and do not try to think about anything. For an anatomic reference, T1 images will be obtained before resting state scans.

The real acupuncture and sham acupuncture groups will be examined three times (before treatment, after treatment, and in the follow-up period.) and the healthy controls will be examined only once.

### Follow-up procedure

Eight weeks after the end of treatment, the PSQI, fMRI, FS-14, HAS, HAMD, actigraphy, and sleep diary records will be collected.

### Outcome measurements

Figures 1 and 2 show all outcome parameters.

### Primary outcome

Primary outcome is the mean change in PSQI scores from baseline to the end of the treatment period. The PSQI, a self-assessment questionnaire to evaluate sleep quality, will be the primary outcome measurement [25]. The PSQI contains 19 items consisting of seven component scores, including sleep quality, sleep latency, sleep duration, daytime dysfunction, sleep efficiency, sleep disturbances, and sleeping medication. Higher global scores reflect worse sleep quality [26].

### Secondary outcomes

#### Sleep diaries

Sleep diaries will be assessed for sleep quality [27]. Participants with insomnia disorder will be required to record their bedtime, sleep time, wake-up time, sleep latency, the state after morning awakening, and related factors in their sleep diaries. Recording of sleep diaries will be performed at baseline and at 4 and 12 weeks following entry into the study.

#### Hyperarousal scale

The HAS is a self-evaluation questionnaire containing 26 items. The questionnaire will be employed to evaluate the behaviors of cortical arousal, including information processing, strong response to unexpected stimuli, and introspective tendencies [28]. The scores correlate positively with a heightened arousal state. This questionnaire is usually applied to measure the change in increased cortical responsiveness [29].

#### Fatigue scale-14

Fatigue is a dominant effect of insomnia [30]. The FS-14 is a self-rating scale to measure the severity of physical and mental fatigue. The FS-14 scores correlate positively with fatigue severity.

#### HAMD and HAMA

Assessment of depressive status will be made with the HAMD. The HAMD is comprised of 17 variables with five- or three-point scales. Scores will be interpreted as follows: less than 7, normal; 8 to 17, mild depression; 17 to 24, moderate depression; and 24 or over, severe depression [31].

The 14-item HAMA will be used to determine anxiety. The HAMA is comprised of a 14-point self-assessment questionnaire, with a five-point scale for each item. A normal score is less than 7, scores between 7and 14 suggest mild anxiety, scores between 14 and 20 indicate moderate anxiety, scores between 21 and 28 indicate severe anxiety, and a score greater than or equal to 29 indicates extremely severe anxiety [32].

For patients with insomnia disorder, the scores for HAMD and HAMA will be collected at baseline, week 4, and at the 8-week follow-up. The HAMD and HAMA will be collected only once at baseline in the healthy control group. Collection of the week-long sleep diaries will be at baseline, at 4 weeks after treatment, and at the end of the 8-week follow-up.

#### Actigraph

To obtain objective data on sleep and activity, we will request that participants wear an actigraph (MTI Health Services Company, Pensacola, FL, USA) on their nondominant wrist for three 1-week periods (before the intervention, at the end of the intervention period, and at the end of the follow-up period) [33].

#### Safety monitoring

Potential adverse events from acupuncture include fainting, needle sticking, infections at the puncture site, and subcutaneous hematoma. Participants will be asked to report adverse events, and practitioners will report any adverse events at each patient visit. Vital signs and adverse events will be chronicled in the case report form at each visit. There is no anticipated harm and no compensation for trial participation.

#### Quality control

To ensure a standard operation procedure, acupuncturists will consistently apply acupuncture with the correct acupoints and manipulation. Investigators will attend training to properly apply the randomization number table, to make the diagnoses, to understand the inclusion and exclusion criteria, and to complete the case report forms. In addition, the fMRI scan will be performed with the same scanner and operator. Patients will be asked to close their eyes during the entire scanning procedure and stay still but not sleep.

Additionally, the Scientific Research Supervision group of the Beijing Hospital of Traditional Chinese Medicine will monitor the study regularly to ensure adequate recruitment rate, data accuracy, and data validity.

### Statistical analysis

#### Clinical data analysis

Statistical analyses will be performed with Statistical Package for the Social Sciences (version 19.0) based on the intention-to-treat principle. Two-sided tests will be considered significant at 5%. We will investigate the reasons for any missing data and, if necessary, multiple imputations will be applied. Data will be evaluated in agreement with the intention-to-treat principle. Sociodemographic information will be shown as the mean ± standard deviation or the frequency (%) when appropriate. The baseline continuous, dependent variable data will be compared using independent-sample *t* tests and chi-square tests will be performed for categorical data. Differences between group means will be analyzed with a repeated-measures analysis of variance. Multivariate analysis of variance will be performed and Bonferroni correction will be applied for pairwise comparisons of changes in questionnaire scores with repeated measurement times for each group.

#### Functional MRI data analysis

The fMRI data will be analyzed with SPM12 software (Wellcome Department of Imaging Neuroscience, London, UK), MATLAB_R2018a (Mathworks, Inc., Natick, MA, USA), DPARSF_V2.2 (http://www.rfmri.org/DPABI), and Freesurfer (http://surfer.nmr.mgh.harvard.edu/) software. Original data will be corrected; slice timing, affine head motion, and nonbrain extraction, spatial smoothing and temporal filtering will be applied.

Baseline ReHo and amplitude of low-frequency fluctuations (ALFF) values and functional connectivity maps from the real and sham acupuncture groups and the healthy control group will be compared using independent *t* tests. Independent *t* tests will be used for comparison of ReHo or ALFF values from the two groups after acupuncture. Paired *t* tests will be used to compare the pre-acupuncture and post-acupuncture data within each group. Average time series data from significant areas will be extracted to test the regional relationship with the rest of the brain using voxel-based general linear modeling. Fisher’s R-to-Z transformation will be applied to compare resting state functional connectivity using independent-sample *t* tests between groups [34].

Pearson’s correlation analyses will be performed to evaluate the relationship of clinical symptoms with ReHo or ALFF values in regions demonstrating significant differences between groups.

## Discussion

The purposes of the trial are to determine the effectiveness of acupuncture for the treatment of insomnia disorder and explore the potential neural mechanisms by which acupuncture affects sleep quality using neuropsychological measurements and rs-fMRI methods.

Based on the hyperarousal hypothesis, we will investigate the effect of acupuncture on spontaneous activities and functional connections in abnormal brain regions. The hyperarousal state was not assessed with fMRI analyses in previous clinical trials. To the best of our knowledge, this is the first randomized controlled trial associating the effects of acupuncture on insomnia disorder with its effects on brain hyperarousal state measured with rs-fMRI methods. The trial will provide important data on the effect of acupuncture and its possible mechanistic associations.

The influence of acupuncture on spontaneous regional brain activity and functional connectivity in patients with insomnia disorder will be explored using ALFF, ReHo, and functional connectivity analyses. ALFF [35] and ReHo analyses are simple, easily implemented methods which exhibit favorable test–retest reliability and have been successfully applied to insomnia research. Increased information processing in the emotion-related regions, manifested as higher ReHo and ALFF scores, suggest an association with the hyperarousal state of insomnia disorder [13–16]. However, the results of the previous studies have not been consistent enough to yield any general conclusions. Thus, we included a healthy control group. Baseline ReHo and ALFF values and functional connectivity maps from the patients with insomnia and the healthy control group will be compared to explore the characteristics of brain regions in patients with insomnia disorder.

In a recent study, acupuncture therapy decreased hyperarousal level and improved sleep quality in patients with insomnia disorder [9]. We speculate that this effect may have relevance to the modulation of regional brain activity and functional connectivity, especially in emotion-related areas. If our hypothesis is correct, ALFF and ReHo values and functional connectivity maps of the emotion-related regions will be modulated more in the group receiving acupuncture than in the sham group. These results will provide insight into the potential intrinsic mechanism of acupuncture for treating insomnia disorder.

Long-term effectiveness is an important feature of the ideal treatment for insomnia. We found that verum acupuncture exhibited long-term effects in the follow-up period compared with sham acupuncture in patients with insomnia [23]. The possible neurobiological basis of the long-term effects is not well understood. Acupuncture may induce a long-term regulatory effect on brain function. In our trial, all patients will undergo fMRI during the follow-up period. One of the greatest strengths of the present study is the investigation of the long-lasting effects of acupuncture on insomnia and the central mechanism of the long-term effects of acupuncture.

Actigraphy will be applied in our trial to assess the changes in sleep quality. Although actigraphy may not be as accurate as polysomnography, this method can provide an accurate estimate of typical sleep duration. Actigraphy is more accepted and well tolerated by patients with insomnia disorder. Furthermore, actigraphy can record sleep in the patients’ natural environment and decreases the interference of factors that change a patient’s characteristic sleep patterns [36]. Meanwhile, sleep diaries and questionnaires will be recorded at the same time as the actigraphy. At follow-up, rs-fMRI and actigraphy analyses will be performed to provide objective measures related to the long-term alterations of sleep quality and brain function in insomnia disorder.

The design of an appropriate control group for a clinical trial is critical. However, it is difficult to use placebo needles for controls. Thus, we will use acupoints in the sham acupuncture group that are considered ineffective for insomnia according to our clinical experience. The possible nonspecific physiological reactions induced by needle pricking will be minimized by superficial puncturing and no manipulation to avoid the De qi sensation, which is believed to be fundamental to the efficacy of acupuncture.

The acupuncturists will receive training for consistent practices, including acupuncture point location and manipulation, to achieve effective quality control.

Despite the measures taken to ensure a well-controlled trial, there are several methodologic limitations to this study. First, the treatment intervention duration of 4 weeks may be insufficient to achieve a measurable response, and a prolonged treatment period may result in a better effect. Second, there is no insomnia subgroup design. According to the definition of psychophysiological insomnia, this subtype has more relevance to the hyperarousal state with learned sleep-preventing associations [37].. Other subtypes such as paradoxical insomnia and adjustment insomnia may have lower levels of hyperarousal. In our study, the insomnia disorder inclusion criteria of a “score above 32 on the HAS” will ensure the degree of hyperarousal. In further research, subgroup design and prolonged treatment periods should be considered when investigating the efficacy of acupuncture in treating insomnia. With the improvements in fMRI techniques, further advances can be made to clarify the neural substrates underlying the effects of acupuncture in insomnia disorder. In spite of its limitations, we hope our trial will help to provide new insights into the central neurological basis of acupuncture for treating insomnia disorder.

## Trial status

The Medical Ethical Committee of the Beijing Hospital of Traditional Chinese Medicine approved the study protocol on 12 March 2018 (permission 2018BL-002-02 for protocol 20,180,209). This trial was registered in March 2018 (ChiCTR1800015282). Due to the time required for recruitment and preparation, the trial started in November 2018. The first participant was included in January 2019, and six participants have been recruited as of the date of this submission. The trial is currently recruiting participants. We predict that recruitment will be completed by November 2020.

## Supplementary information

**Additional file 1:** SPIRIT 2013 checklist: Recommended items to address in a clinical trial protocol and related documents*.

## Data Availability

All study-related data will be stored securely at the Beijing Hospital of Traditional Chinese Medicine. The datasets analyzed during the current study are available from the corresponding author for a future secondary analysis.

## Abbreviations

ALFF: Amplitude of low-frequency fluctuations
FS-14: Fatigue scale-14
HAMA: Hamilton anxiety scale
HAMD: Hamilton depression scale
HAS: Hyperarousal scale
PSQI: Pittsburgh Sleep Quality Index
ReHo: Regional homogeneity
rs-fMRI: Resting state functional magnetic resonance imaging
